# Do large-scale hospital- and system-wide interventions improve patient outcomes: a systematic review

**DOI:** 10.1186/1472-6963-14-369

**Published:** 2014-09-03

**Authors:** Robyn Clay-Williams, Hadis Nosrati, Frances C Cunningham, Kenneth Hillman, Jeffrey Braithwaite

**Affiliations:** Centre for Clinical Governance Research, Australian Institute of Health Innovation, University of New South Wales, Level 1, AGSM Building, Sydney, NSW 2052 Australia; The Simpson Centre for Health Services Research, Australian Institute of Health Innovation, University of New South Wales, Sydney, Australia; Menzies School of Health Research, Brisbane, Australia

**Keywords:** Organisational culture, Patient outcomes, System implementation, Hospital-wide intervention, Patient safety, Implementation science, Hospital care

## Abstract

**Background:**

While health care services are beginning to implement system-wide patient safety interventions, evidence on the efficacy of these interventions is sparse. We know that uptake can be variable, but we do not know the factors that affect uptake or how the interventions establish change and, in particular, whether they influence patient outcomes. We conducted a systematic review to identify how organisational and cultural factors mediate or are mediated by hospital-wide interventions, and to assess the effects of those factors on patient outcomes.

**Methods:**

A systematic review was conducted and reported in accordance with Preferred Reporting Items for Systematic Reviews and Meta-Analyses (PRISMA) guidelines. Database searches were conducted using MEDLINE from 1946, CINAHL from 1991, EMBASE from 1947, Web of Science from 1934, PsycINFO from 1967, and Global Health from 1910 to September 2012. *The Lancet*, *JAMA*, *BMJ*, *BMJ Quality and Safety*, *The New England Journal of Medicine* and *Implementation Science* were also hand searched for relevant studies published over the last 5 years. Eligible studies were required to focus on organisational determinants of hospital- and system-wide interventions, and to provide patient outcome data before and after implementation of the intervention. Empirical, peer-reviewed studies reporting randomised and non-randomised controlled trials, observational, and controlled before and after studies were included in the review.

**Results:**

Six studies met the inclusion criteria. Improved outcomes were observed for studies where outcomes were measured at least two years after the intervention. Associations between organisational factors, intervention success and patient outcomes were undetermined: organisational culture and patient outcomes were rarely measured together, and measures for culture and outcome were not standardised.

**Conclusions:**

Common findings show the difficulty of introducing large-scale interventions, and that effective leadership and clinical champions, adequate financial and educational resources, and dedicated promotional activities appear to be common factors in successful system-wide change.

The protocol has been registered in the international prospective register of systematic reviews, PROSPERO (Registration No. CRD42103003050).

**Electronic supplementary material:**

The online version of this article (doi:10.1186/1472-6963-14-369) contains supplementary material, which is available to authorized users.

## Background

Patient safety, quality improvement and implementation science have become major foci of change activities [1–5]. As understanding of the complex nature of the healthcare system grows [6, 7], the importance of system-level change is emerging [8]. Attempts to introduce cross-organisational interventions such as the Medical Emergency Team (MET) have met with mixed success [9], and initiatives at an organisational level, which include participative principles such as the involvement of clinicians in improvements [10, 11], may provide the greatest hope of realising productive change [12]. However depending on the presence of necessary infrastructure to ensure good standards of care [13, 14], the effectiveness of hospital-wide interventions can vary significantly from one health organisation to another [9]. Few system-wide interventions have been implemented in acute hospitals. Most work has been in the form of local change, centred on quality improvement and patient safety initiatives that have shown limited patient benefits [15], with typical effect sizes of perhaps 10-20% at best [16].

We know that there is variability in uptake of system-wide healthcare interventions, [9, 17, 18] but we do not fully appreciate how organisations and cultures affect interventions or how organisational factors affect uptake [19–23]. Nor, despite much discussion, do we understand the sustainability of hospital-wide interventions, or how implementation creates change or influences patient outcomes in acute hospitals and other parts of health care. A systematic review on this topic may allow generalisations to be made on the efficacy of large-scale interventions that could inform future implementation of these strategies for improving safety. This review aims to investigate large-scale system change by identifying how organisational and cultural factors [22, 24] mediate or are mediated by hospital- and system-wide interventions, and by assessing the effects of those factors on patient outcomes.

## Methods

The protocol for this review has been registered in the international prospective register of systematic reviews, PROSPERO (Registration No. CRD42103003050). The protocol was published in *BMJ Open*[25], and the full text can be accessed at: http://bmjopen.bmj.com/content/3/3/e002268.full?sid=819c5b6d-ca14-4f6f-81ba-308d99da2870.

### Search strategy

We searched MEDLINE, CINAHL, EMBASE, Web of Science, PsycINFO, Global Health, and Scopus from 1946, 1991, 1947, 1934, 1967, 1910, respectively to September 2012, using Medical Subject Headings and keywords. The full search strategy is shown in Additional file 1: Table A. Multiple terms were used to identify culture and intervention. Although we restricted the search to English language articles, a recent systematic review of empirical studies on the effect of restricting studies to English language found no evidence of systematic bias associated with this procedure in systematic reviews in conventional medicine [26]. To check that database searches did not miss any relevant studies, and to confirm the suitability of our search criteria, we hand searched the journals *The Lancet*, *JAMA*, *BMJ*, *BMJ Quality and Safety*, *The New England Journal of Medicine* and *Implementation Science* individually for articles between July 2007 to September 2012. These peer-reviewed journals were chosen in terms of likelihood of meeting inclusion criteria, in particular validated patient outcomes. We also hand searched the reference lists of the Cochrane systematic reviews that were identified in the primary search. We did not include ‘grey literature’ as it was unlikely to yield study designs that met inclusion criteria.

### Study selection and exclusion criteria

**Figure 1:**
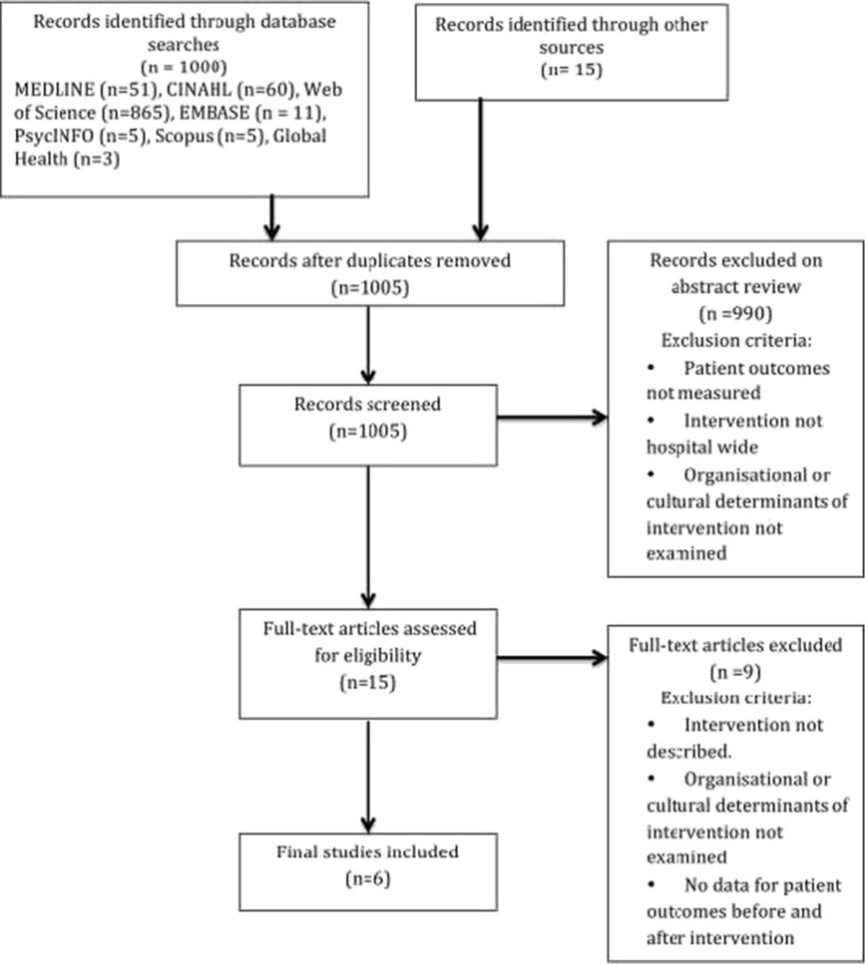
**Systematic review flowchart.**

Figure 1 shows the process by which studies were selected for review. Our initial search yielded 1000 articles. The hand search yielded another 15 papers, giving a total of 1015 articles. After the removal of duplicates (n = 10), two reviewers (HN and RCW) independently undertook a title and abstract review of the remaining 1005 articles using the inclusion and exclusion criteria shown in Figure 1.

Inclusion and exclusion criteria required empirical, peer-reviewed articles in English where the abstract and full title were available. As we sought hospital-wide interventions, those limited to operating theatres, wards and units or ICUs were excluded. Other inclusion criteria required that the study investigate organisational factors that might influence or be influenced by the implementation. Studies had to provide patient outcome data before and after an intervention that was hospital-wide and in an acute care setting, i.e. rehabilitation centres, primary health care, ambulatory services, and psychiatric facilities were excluded.

These criteria yielded 15 articles, which were obtained in full text for independent assessment by two reviewers. Study authors were contacted for further information if necessary. Studies were excluded only after discussion between three reviewers (HN, RCW, FC), who assessed and agreed on the inclusion and quality rating of the studies. From this review we derived six studies that met all inclusion criteria.

### Data extraction

One author (HN) extracted summary descriptive data and compiled a tabular presentation of the study participants and setting, objectives, design and method, type of hospital-wide intervention, organisational and cultural factors, patient and process outcomes, and findings. A second author (RCW) reviewed this documentation for accuracy and completeness. Published supplementary documents [27–30] to four articles [31–34] were reviewed in addition to the primary articles, and the authors of one study [35] were contacted, and provided additional information.

### Assessment of risk of bias

Two authors (HN and RCW) independently assessed risk of bias. They applied standard Cochrane criteria [36] including selection and allocation biases, potential confounders, blinding, reliability of the outcome measures and baseline comparisons.

## Results

### Classification of interventions

We classified the interventions into three categories consisting of hand hygiene, multifaceted patient safety interventions and electronic health record. Additional file 1: Table B provides a summary of each intervention, including details of study participants and settings, objectives, design and method, type of hospital-wide intervention, organisational and cultural factors, patient and process outcomes, and findings.

### Characteristics of studies

Table 1 provides an overview of the studies. Of the six studies directed at hospital-wide interventions, four had hand hygiene either as the only intervention (Larson, Grayson), or as a component of the intervention (Lilford1, Lilford2), and three studies had multi-component patient safety initiatives aimed at reducing adverse events, improving monitoring of vital signs, or improving the culture of safety and quality of care (Lilford1, Lilford2, Muething). One study (Nowinski) researched the effects of implementation of an electronic health record system in three hospitals on the organisational culture of those hospitals. Table 2 summarises the key study characteristics. Four of the studies were published between 2011 and 2012. Five out of six were undertaken in the United States (US) (Larson, Nowinski, Muething) or United Kingdom (UK) (Lilford1, Lilford2). The largest study setting – representing 512 hospitals – was in Australia (Grayson).

**Table 1:** **Overview of studies**

Study	Authors	Date	Title	Type
Larson [37]	Larson EL, Early E, Cloonan P, Sugrue S, Parides M.	2000	An organizational climate intervention associated with increased handwashing and decreased nosocomial infections	Non-randomised controlled trial
Nowinski [35]	Nowinski CJ, Becker SM, Reynolds KS, Beaumont JL, Caprini CA, Hahn EA, Peres A, Arnold BJ.	2007	The impact of converting to an electronic health record on organisational culture and quality improvement	Observational study
Grayson [33]	Grayson ML, Russo PL, Crulckshank M, Bear JL, Gee CA, Hughes CF, Johnson PD, McCann R, McMillan AJ, Mitchell BG, Selvey CE, Smith RE, Wilkinson I	2011	Outcomes from the first 2 years of the Australian National Hand Hygiene Initiative	Observational study
Lilford1 [32]	Benning A, Ghaleb M, Suokas A, Dixon-Woods M, Dawson J, Barber N, Franklin BD, Girling A, Hemming K, Carmalt M, Rudge G, Naicker T, Nwulu U, Choudhury S, Lilford R	2011	Large scale organisational intervention to improve patient safety in four UK hospitals: mixed method evaluation	Controlled before and after study
Lilford2 [31]	Benning A, Dixon-Woods M, Nwulu U, Ghaleb M, Dawson J, Barber N, Franklin BD, Girling A, Hemming K, Carmalt M, Rudge G, Naicker T, Kotecha A, Derrington MC, Lilford R	2011	Multiple component patient safety intervention in English hospitals: controlled evaluation of second phase	Controlled before and after study
Muething [34]	Muething SE, Goudie A, Schoettker PJ, Donnelly LF, Goodfriend MA, Bracke TM, Brady PW, Wheeler DS, Anderson JM, Kotagal UR.	2012	Quality improvement initiative to reduce serious safety events and improve patient safety culture	Observational study

**Table 2:** **Characteristics of studies**

Characteristic	Number of studies
*Year*	
2000-2007	1
2007-2011	1
2011-2012	4
*Country*	
United States	3
Australia	1
United Kingdom	2
*Settings (# hospitals)*	
1-4	4
5-20	1
21-100	0
>100	1
*Type of hospital-wide intervention*	
Hand hygiene	4
Electronic health record	1
Multi-faceted patient safety*	3
*Study design*	
Non-randomised controlled trial	1
Observational study	3
Controlled before and after study	2
*Type of patient outcome*	
Mortality	2
Adverse events	4
Patient satisfaction	3
Nosocomial infections	3
Quality Improvement (QI) indicators**	3
*Data collection method*	
Survey	5
Ethnography	1
Case note review	2
Administrative data	6
Interview	1
Study specific data	4

^*^Multi-faceted patient safety intervention might include multiple interventions.

^**^Initial antibiotic dose within 4 h of hospital arrival for pneumonia patients, fall rate per 1000 patient-days, chest pain pathway-discharged within 23 h of admission, annual HgA1c measurement in diabetic patients, left ventricular function evaluation on a yearly basis, appropriate use/non-use of ACE inhibitors [35].

With respect to patient outcomes, Larson, Lilford2 and Grayson measured infection rates; Nowinski, Lilford1, Lilford2 and Muething examined adverse events; Lilford1, and Lilford2 assessed mortality rates; and Nowinski, Lilford1, and Lilford2 evaluated patient satisfaction before and after the intervention. The majority of the studies were observational or controlled before and after design, except the Larson quasi-experimental trial of a hand hygiene intervention. Principal data collection methods included administrative data (all) and survey (all, except Grayson). In addition, two studies used case note review (Lilford1, Lilford2), and Lilford1 also used ethnographic observation and interviews. The studies that focused on hand hygiene and quality improvement initiatives collected specific data for hand hygiene compliance rates (Larson, Grayson), and Serious Safety Events (SSEs) (Muething). The duration of the implementation and the time of follow-up varied amongst studies, from six months (Larson) to four years (Muething).

### Risk of bias in included studies

Of the three controlled studies, Larson had unclear risk of selection bias - allocation to the intervention was not randomised, and the criteria for selecting the intervention group were not indicated. The other two controlled studies (Lilford1, Lilford2) positively selected intervention hospitals based on their likelihood of achieving program success, which resulted in a moderate risk of selection bias. For the remaining three studies, although all participants were subject to the intervention there were no data on attrition over the period of study. The studies generally presented reliable outcomes, analytical analysis, and risk adjustment or a comparison of baseline characteristics. Although none of the studies specifically introduced processes to prevent protection against contamination, only the multifaceted patient safety interventions (Lilford1, Lilford2, Muething) had high risk of contamination from similar patient safety interventions occurring concurrently with the study intervention. Risk of bias associated with the studies is summarised in Additional file 1: Table C.

### Layout of findings

For each of the studies, we extracted data on patient outcomes (clinical indicators and process outcomes) and organisation factors. We then looked for correlations between the patient outcomes and organisation factors. Table 3 provides a summary of the patient outcomes, organisation factors, and correlation between the two. Detailed findings are reported under each of these category headings in the following paragraphs.

**Table 3:** **Key findings for organisational-wide interventions**

Extracted organisational factors	Interventions	Patient outcomes	Process outcomes	Organisational factors correlated with patient outcomes
Staff morale and organisational climate [31, 32]	Multi-faceted patient safety	Improved monitoring of vital signs [32]	Significant improvement in one measure of staff perception of organisational climate (p < 0.01) [32]	None reported
		Significant improvement in one measure of patient satisfaction (cleanliness of the bathrooms) in the intervention hospitals [32].		
			Significant decrease in one measure of staff perception of organisational climate (p < 0.01) [31]	
Organisational culture [35]	Electronic health record	Decrease of 16% in Clinical quality indicator (CQI) for initial antibiotic dose within 4 h of hospital arrival for pneumonia patients (p < 0.001) between intervention and follow-up. Decrease of 3% in CQI for chest pain pathway-discharged within 23 h of admission (p < 0.023) for one of three hospitals between intervention and follow-up [35].	Least-squares adjusted means for group culture decreased from 21.8 to 20.0 after 12 months [35]	Several strong (>0.94) correlations between changes in culture scores and changes in quality indicators at three acute care facilities [35]
			Least-squares adjusted means for hierarchical culture increased from 30.0 to 31.9 after 12 months (change only significant in one of five hospitals for group culture and two of five hospitals for hierarchical culture) [35]	
				Appropriate discharge of patients with chest pain negatively correlated with developmental culture [35]
				Use of antibiotics within 4 h of admission positively associated with rational culture and quality management, and negatively related to group culture and human resource utilisation [35]
		*Decreased patient satisfaction for two of three hospitals between intervention and follow-up (1*%*, p < 0.003 and 2*%*, p < 0.019)*[35]*.*		
				Patient satisfaction positively correlated with group culture and negatively correlated with rational culture [35]
Patient safety culture [33, 34, 37]	Hand hygiene	National incidence rates of methicillin resistant SAB were stable for the 18 months prior to NHHI (Jul 2007–2008; p = 0.366) but declined after implementation (2009–2010; p = 0.008) [33]	For sites new to ‘5 Moments’ audit tool, hand hygiene compliance increased from 43.6% to 67.8% after 2 years (P < 0.001) [33]	None reported
	Multi-faceted patient safety			
		Reduction in nosocomial infections associated with MRSA and VRE in the intervention hospital between baseline and follow up phases for were both significantly greater than change in comparison hospital (P < 0.0001) [37]	Frequency of hand washing in study hospital was more than double that in control at 6 month follow-up [37]	
			During initial phase of the interventions, results from safety culture survey worsened. However, as initiative progressed, there was improvement [34]	
		Following the intervention, SSEs per 10,000 adjusted patient days significantly decreased from a mean of 0.9 to 0.3 (p < 0.0001). Days between SSEs increased from a mean of 19.4 to 55.2 (p < 0.0001) [34]		
Organisational and clinical Leadership [31–35, 37]	Multi-faceted patient safety	Improved monitoring of vital signs [32]	Least-squares adjusted means for leadership showed decrease in the leadership scale after 12 months of electronic health record implementation from 3.63 to 3.54, but only significant (p < 0.05) in one of five hospitals [35]	None reported
	Hand hygiene	Significant improvement in one measure of patient satisfaction (cleanliness of the bathrooms) in the intervention hospitals [32].		
	Electronic health record			
		National incidence rates of methicillin resistant SAB were stable for the 18 months prior to NHHI (Jul 2007–2008; p = 0.366) but declined after implementation (2009–2010; p = 0.008) [33]		
		Following the intervention, SSEs per 10,000 adjusted patient days significantly decreased from a mean of 0.9 to 0.3 (p < 0.0001). Days between SSEs increased from a mean of 19.4 to 55.2 (p < 0.0001) [34]		
		Decrease of 16% in Clinical quality indicator (CQI) for initial antibiotic dose within 4 h of hospital arrival for pneumonia patients (p < 0.001) between intervention and follow-up. Decrease of 3% in CQI for chest pain pathway-discharged within 23 h of admission (p < 0.023) for one of three hospitals between intervention and follow-up [35].		
		Decreased patient satisfaction for two of three hospitals between intervention and follow-up (1%, p < 0.003 and 2%, p < 0.019) [35].		
		Reduction in nosocomial infections associated with MRSA and VRE in the intervention hospital between baseline and follow up phases for were both significantly greater than change in comparison hospital (P < 0.0001) [37]		
Education, training and assessment [31–35, 37]	Multi-faceted patient safety	Improved monitoring of vital signs [32]	Standardised hand hygiene ‘5 moments’ auditing tool and audit training implemented across hospitals [33]	None reported
	Hand hygiene			
	Electronic health record			
		Significant improvement in one measure of patient satisfaction (cleanliness of the bathrooms) in the intervention hospitals [32].	Least-squares adjusted means for human resources utilisation after 12 months of electronic health record implementation increased for two of the five hospitals (from 3.05 to 3.18 and from 3.38 to 3.57, respectively (P < 0.05)) [35]	
		National incidence rates of methicillin resistant SAB were stable for the 18 months prior to NHHI (Jul 2007–2008; p = 0.366) but declined after implementation (2009–2010; p = 0.008) [33]		
		Decrease of 16% in Clinical quality indicator (CQI) for initial antibiotic dose within 4 h of hospital arrival for pneumonia patients (p < 0.001) between intervention and follow-up. Decrease of 3% in CQI for chest pain pathway-discharged within 23 h of admission (p < 0.023) for one of three hospitals between intervention and follow-up [35].		
		Decreased patient satisfaction for two of three hospitals between intervention and follow-up (1%, p < 0.003 and 2%, p < 0.019) [35].		
		Reduction in nosocomial infections associated with MRSA and VRE in the intervention hospital between baseline and follow up phases for were both significantly greater than change in comparison hospital (P < 0.0001) [37]		
Promoting and awareness of the intervention [33, 34]	Multi-faceted patient safety	National incidence rates of methicillin resistant SAB were stable for the 18 months prior to NHHI (Jul 2007–2008; p = 0.366) but declined after implementation (2009–2010; p = 0.008) [33]	None reported	None reported
	Hand hygiene			
		Following the intervention, SSEs per 10,000 adjusted patient days significantly decreased from a mean of 0.9 to 0.3 (p < 0.0001). Days between SSEs increased from a mean of 19.4 to 55.2 (p < 0.0001) [34]		

### The effects of the hospital-wide intervention on patient outcomes

Four of the six studies found significant improvement in patient outcomes associated with the intervention (Larson, Nowinski, Grayson, and Muething). In Larson, the mean hand-washing frequency per patient-care day at six-months follow up in the study hospital was double that of the comparison hospital. At baseline there were no significant differences between rates of VRE and MRSA, in the study and comparison hospitals. The study hospital showed significantly lower rates in terms of VRE both in implementation and follow-up phases, but not significant differences in MRSA in those phases. The ratio of change (i.e. the reduction in infection rates) in the intervention hospital between baseline and follow up phases for MRSA and VRE were both significantly greater than the ratios of change in comparison hospitals.

The Australian National hand hygiene program (Grayson) reported the overall national HH compliance rate in 521 hospitals was 68.3% (168 641/246 931 moments) in late 2010, but HH compliance before patient contact was 10%–15% lower than after patient contact. Among sites new to the “5 Moments” audit tool, HH compliance improved from 43.6% (6431/14740) at baseline to 67.8% (106 851/157 708) (p < 0.001). Educational programs appear to have influenced professional groups differently. HH compliance was highest among nursing staff (73.6%; 116 851/158 732) and lowest among medical staff (52.3%; 17 897/34 224) after two years. National incidence rates of Methicillin-Resistant Staphylococcus Aureus Bacteraemia (MRSAB ) were stable for the 18 months before the NHHI (July 2007–2008; p = 0.366), but declined after implementation (2009–2010; p = 0.008). Annual national rates of hospital-onset Staphylococcus Aureus Bacteraemia (SAB) per 10 000 patient-days were 1.004 and 0.995 in 2009 and 2010 respectively, of which about 75% were due to MRSAB.

In Nowinski, three quality indicators across two of the three acute hospitals in the study had changes from Baseline to Time 2 (one year after intervention) that were statistically significant. The percentage of patients with pneumonia receiving initial antibiotic dose within four hours of hospital arrival decreased from 95 to 79% (p < 0.001) for the network as a whole, with each site also showing significant decreases. Discharge of chest pain patients within 23 hours of hospital arrival also decreased from 94 to 91% (p < 0.023). Patient satisfaction worsened in two of the three hospitals, decreasing 2% (p < 0.019) for one hospital and 1% (p < 0.003) for another. There were several strong (>0.94) correlations between changes in culture scores and changes in quality indicators at the three acute care facilities. Appropriate discharge of patients with chest pain was negatively correlated with developmental culture; use of antibiotics within four hours of admission was positively associated with rational culture and quality management and negatively related to group culture and human resource utilisation; and patient satisfaction was positively correlated with group culture and negatively correlated with rational culture.

Lilford1 found the intervention was associated with improvements in one of the types of clinical process studied (monitoring of vital signs) and one measure of staff perceptions of organisational climate. There was no additional effect of the processes on other targeted issues or on other measures of generic organisational strengthening.

The outcome results for Lilford2, as for Lilford1, suggested that it was difficult to detect any additive intervention effect. No significant change in terms of adverse events and adjusted mortality rate between control and intervention hospitals was detected. Only one of the patient satisfaction scores (cleanliness of the bathrooms) showed significantly different change favouring the intervention hospitals [32]. Lilford2 found the intervention was associated with change in one measure of staff perceptions of organisational climate but, unlike in Lilford1, this change favoured the control hospitals.

The outcome results within four years following the quality improvement intervention across the CCHMC (Muething) showed the number of SSEs per 10,000 adjusted patient-days significantly decreased from a mean of 0.9 to 0.3 (p = 0.001). Days between SSEs increased from a mean of 19.4 to 55.2 (p = 0.001). During this same time, patient volume increased; monthly average adjusted patient-days were 13,686 during the baseline period and 17,521 during the study period. The reduction in SSEs occurred gradually but then seemed to stabilise at 0.3 SSEs per 10,000 adjusted patient-days. The authors of the study believe this result reflected a combined effect from system improvements and cultural change.

### Organisational factors

The concepts of organisational culture, organisational climate and patient safety culture overlapped and were not commonly defined. Thus, it was difficult to establish the organisational factors as a set of discrete variables. Organisational determinants were identified in the six studies as (1) staff morale and organisational climate; (2) organisational culture; (3) patient safety culture; (4) clinical and organisational leadership; (5) education, training and assessment; and (6) promotion and awareness of the intervention.

### Staff morale and organisational climate

Of the six studies, the two studies evaluating the UK Health Foundation’s Safer Patients Initiative (SPI) (Lilford1 and Lilford2) examined staff morale and opinion, and organisational climate. Organisational climate refers to the extent of positive feeling within the organisation relating to communication, staff involvement, innovation and patient care [38, 39]. Thirteen of 28 survey items in the National Health Service (NHS) national staff survey, encapsulating staff morale and organisational climate, were completed before and after the intervention in the control and intervention hospitals. Surveyed items consisted of working in well-structured teams, job satisfaction, quality of work-life balance; support from supervisors, and organisational climate. Only one of the 11 (Lilford1), and 13 (Lilford2) items – the score for organisational climate – was found to have a statistically significant (p < 0.01) change over time between the control and intervention hospitals. Improvement in organisational climate favoured the intervention hospitals in Lilford1 and the control hospitals in Lilford2 [31, 32].

### Organisational culture

Nowinski examined four types of organisational culture – group or clan, rational, hierarchical and developmental – based on the Competing Values Framework (CVF) [40] before and after the intervention. Organisational culture was categorised along three dimensions: focus on people versus the organisation; preference for structure; and emphasis on specific type of strategies and outcome [35, 39]. Organisations are likely to be a combination of each of these culture types, however, the study found that the culture was perceived to be more hierarchical following implementation of the intervention.

### Patient safety culture

Muething focused on improving the patient safety culture while trying to reduce SSEs in a large urban pediatric hospital. Safety culture was assessed using the US Agency for Healthcare Research and Quality (AHRQ) Hospital Survey on Patient Safety Culture. The survey measured seven unit-level dimensions of safety culture (teamwork within units, supervisor/manager expectations and actions promoting patient safety, organisational learning – continuous improvement, staffing, non-punitive response to errors, feedback and communication about error, and communication openness) and three hospital-level dimensions (teamwork across units, management support for patient safety, and handoffs and transitions) [41]. Patient safety culture outcomes were found to worsen in the first year of the intervention (2006) but subsequently improved between 2007 and 2009.

### Leadership

All six studies found organisational leadership and the presence of clinical intervention champions to be essential elements in a successful implementation. Definitions of leadership were inconsistent, however, and the direct contribution of leadership to outcomes was difficult to determine. Grayson divided leadership into (i) executive commitment, (ii) clinical leadership team, and (iii) staff ownership. Lilford2 noted that leadership was a two faceted concept: even though interviews showed that senior stakeholders were generally enthusiastic and knowledgeable about the proposed processes, there was only modest penetration down to ward level as staff saw the intervention as imposed rather than inclusive. Nowinski proffered an alternate view, and assessed the effects of an electronic health record on hospital leadership as an indicator of the degree of continuous quality improvement (CQI) maturity within the organisation. In Muething, a patient safety oversight group focused on accountability, balancing quick fixes and long-term solutions in response to safety events, allocating organisational resources and quality improvement infrastructure to strategic priorities, and transparency to the organisation and the public.

### Education, training and assessment

All six studies dedicated financial and managerial resources for training and education associated with the intervention; however processes were specific to the organisation. In the Grayson study, Hand Hygiene Australia (HHA) had a well-defined strategy and conducted the training, education and assessment through multiple means: the HHA Website, Auditor Workshops, HHA ‘5 Moments’ program implementation and auditing manual, HHA training DVD, HHA Auditor Training Recommendations, Annual Auditor Validation Recommendations, online learning packages, eBulletin, online data entry, promotional posters, sample hand hygiene policies, product selection and placement recommendations, educational materials, pamphlets, and presentations.

In Lilford1 and Lilford2, 15–20 change-agents from the four UK intervention hospitals each participated in change management learning sessions run by the US Institute for Healthcare Improvement (IHI). Change agents were charged with leading change by facilitating the implementation of the SPI intervention, and by forming a virtual community to share data, expertise and experience. Participating hospitals received support and visits from IHI throughout the course of the program, which lasted for 18 months.

In Larson, an educational session describing the outcomes of engineering, motivational, and behavioural strategies on improving handwashing compliance was presented to 20 selected managers. From this group, six to eight individuals representing the study units and other key departments volunteered to meet with the research group to develop specific strategies, using the information from the larger group’s brainstorming. They reinforced various components of the intervention with unit and departmental leadership, as needed, by making rounds on units, discussing problems of implementation with the responsible nursing leaders, and suggesting ways to resolve these problems.

Education and assessment in Nowinski was different to that in the other studies, in that it involved collaboration between the medical informatics and information system teams. The implementation process was re-designed continuously to improve clinician communication and reduce error rates, and to facilitate quality improvement efforts leading to better patient management and outcomes [35].

Muething conducted education, training and assessment, embedding these in the intervention. Activities included error prevention, restructuring patient safety governance, a root cause analysis program, lessons learned program, and tactical intervention for high-risk areas.

### Promotion and awareness of the intervention

The Grayson hand hygiene study suggested that awareness of the organisational-wide intervention was an important factor to sustain the cultural change towards hand hygiene among healthcare workers. To achieve this aim, multiple promotional activities were introduced [42] including giveaways; social functions; “slogan’’ competitions; quizzes, crosswords and word searches; pay slip notices; internal magazines and newsletters; and screen savers.

In Muething, staff were given access to all the serious safety events information which encouraged transparent and visible feedback to promote the culture of safety. The Cincinnati Children’s Hospital Medical Center’s (CCHMC’s) intranet site, available to all employees, displayed the number of days since the last SSE, safety stories, and a weekly report from the safety officer designed to reinforce expected safety behaviors, increase staff mindfulness and awareness, celebrate successful interventions, and share details of failures.

### Patient process and outcome indicators

We categorised patient outcomes into five groups: infection rates, quality indicators, adverse events, mortality rates, and patient satisfaction. The interpretation of each outcome measure is explained as follows.

1. *Infection rates*. Larson reported the nosocomial infections associated with MRSA and VRE: the number of cases was divided by the patient-care days and was reported for every 1000 patient-care days. Grayson reported Staphylococcus Aureus Bacteraemia (SAB) incidence rates. In the second phase of SPI, Lilford2 reported the rates of infection with C difficile per 1000 bed occupancy days and MRSA per 100 000 bed occupancy days using routine data from the Health Protection Agency.
2. *Quality Indicators*. Nowinski examined six quality indicators as the primary outcome for the EHR intervention: initial antibiotic dose within four hours of hospital arrival for pneumonia patients; fall rate per 1000 patient-days; chest pain pathway-discharged within 23 hours of admission; annual Hemoglobin A1c (HgA1c) measurement in diabetic patients; left ventricular function evaluation on a yearly basis; and appropriate use/non-use of angiotensin-converting-enzyme (ACE) inhibitors at discharge for patients with Chronic Heart Failure (CHF). These quality indicators, however, are proxy measurements and we do not know if patient outcomes were improved.
3. *Adverse events*. Adverse events (preventable and non-preventable) in Lilford1 and Lilford2 were measured as the incidence of patient harm in six categories: diagnosis/assessment admission error; hospital-acquired infection; technical/management such as a technical problem relating to a procedure and problem in management/monitoring (including nursing and other professional care); medication/maintenance/test results; clinical reasoning; and discharge information. Results were given as total adverse events per 100 patients. Adverse events in the quality improvement initiative [34] were measured as the number of SSEs per 10,000 adjusted patient days and the number of days between SSE occurrence. As in Nowinski, we do not know if these proxy indicators correlated with patient outcomes.
4. *Mortality rates.* Lilford1 and Lilford2 examined mortality among acute respiratory patients aged over 65 (in Lilford1 and Lilford2), and patients in intensive care units whose case notes were selected for review (only in Lilford2). Older patients were selected both because measurement was feasible and, arguably, a higher signal to noise ratio would be expected among this group, which not only benefits from specific SPI interventions but also has high mortality. Routine data from intensive care national audit and research centres in all of the study hospitals were available on a monthly basis for six months before commencement of SPI2 (Lilford2) and for six months after the intervention. Data were available for the numbers of deaths and expected numbers of deaths, which were then used to calculate observed-to-expected mortality ratios. Data were also available on two mean risk prediction scores: the acute physiological and chronic health evaluation (APACHE) II score and the Intensive Care National Audit and Research Centre (ICNARC) score for patients admitted directly from a ward. Despite the ready availability of data, the limitations of using risk adjusted mortality to assess patient safety interventions are well documented [43, 44]. In any case, no significant difference in adjusted mortality rates was found between intervention and control hospitals in either Lilford1 or Lilford2.
5. *Patient Satisfaction.* In Lilford1 and Lilford2, patient views were assessed by means of the NHS survey. An organisational analysis was conducted using a two-way analysis of variance (ANOVA) (the factors being intervention versus control hospital, and survey one versus survey two). Five scores were identified for analysis: three overall satisfaction scores and two related to cleanliness. Organisation-level scores in each arm of the study were determined by averaging all respondents’ scores within each hospital. Nowinski also gathered data on patient satisfaction, and used standard Press Ganey Surveys [45] to assess the effect of the electronic health record.

## Discussion

**Figure 2:**
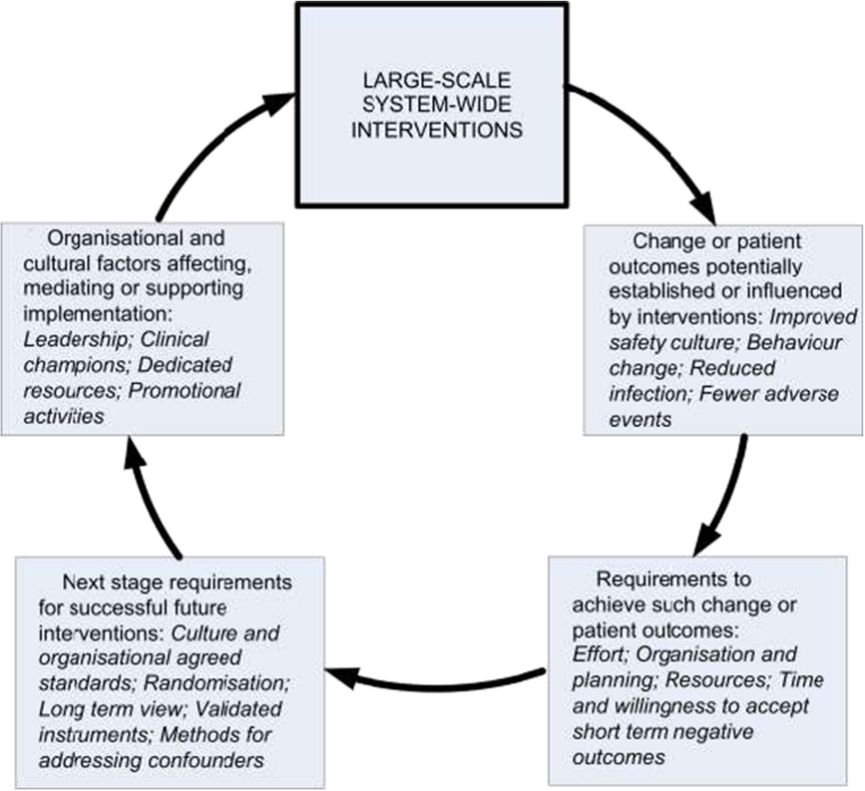
**Factors affecting, and affected by, large-scale system-wide interventions.**

Patient outcomes such as nosocomial infections associated with VRE and MRSAB, monitoring of patients in acute care, and number of SSEs can exhibit improvements associated with such interventions. Enabling these results requires considerable efforts, organisation and resources. Time needed to achieve longer term outcomes is often in excess of the length of the project; further, or sustainable, results can only be realised if the project’s desired outcomes are embedded in the organisation’s culture. Organisations must be willing to accept short term negative outcomes in pursuit of longer term gains. In essence, change at scale is difficult to realise and improved outcomes and measurable effect sizes are hard to deliver. The factors affecting large-scale system-wide interventions in acute settings, and how those interventions establish change and influence patient outcomes, are summarised in Figure 2.

Across these large-scale studies, common organisational factors for successful implementation are leadership and clinical champions, dedicated financial and educational resources and promotion and awareness-raising activities. While each of the studies identifies organisational and clinical leadership as being important, what comprises that ‘leadership’ is not sufficiently well defined in some of the published material to determine which characteristics of leadership the studies have in common. In two of the studies that discussed the importance of executive buy-in [31, 35], the ‘top down’ leadership seemed to have an adverse effect where staff saw the intervention as something that was imposed. This may be partly as a result of the intervention process: in Nowinski [35] where, contrary to expectation, leadership was found to be more hierarchical one year following the intervention, the necessity of applying new rules and processes as a function of introducing the EHR may have fostered a perception of increased management control. In a similar way to leadership, dedicated financial and educational resources, and promotion and awareness-raising activities, are not commonly defined so conclusions cannot be reached regarding their use in future interventions. Economic appraisals or qualitative studies are needed to better answer these questions [46]. Comprehensive studies in the literature on the impact of leadership on successful implementation of interventions suggest that a multi-faceted approach is required, including stated and material support for the intervention at multiple levels of leadership throughout the organisation and increases in levels of trust between leaders and front line clinicians.

No study made the links between the intervention, culture and patient outcomes that would allow a definitive determination to be made in respect of our question. The small number of peer reviewed studies may be an indication that many interventions in hospitals target specific problems or focus on microsystems (e.g. teams congregating around specific clinical diseases, or in individual hospital departments such as ED or ICU) rather than take a system-wide approach. It is difficult to isolate variables such as organisational or cultural determinants and patient outcomes that affect a specific organisation-wide intervention among the clutter of many large and small simultaneous interventions occurring within healthcare systems at any one time. Also, organisation-wide activities sometimes fall under the auspices of ‘quality improvement programs’, and are therefore more likely to be evaluated, when they are evaluated, by an internal quality audit than arm’s length researchers. Where these have been formally evaluated, only modest improvements have been found [15].

As there is no agreed standard or generally accepted protocol for measuring cultural outcomes, it was not possible to compare or combine the studies, or to derive generalisable findings. With the large number and variety of activities occurring in different parts of the intervention hospitals, it was difficult for the researchers to control for confounders in their studies. Selection bias was evident throughout, as intervention groups were not randomised; in some cases, such as Lilford1 and Lilford2, groups were specifically selected with success in mind. Timing appeared to be a factor: most studies were constrained by time, and data were typically gathered within 12 months of the intervention. While it is recognised that culture is a slow changing phenomena [47], the nature of research programs necessitated taking measures quickly. Indications from at least one of the studies [31], and from other healthcare implementation research [11], however, are that changes may not have had sufficient time to mature. Improved outcomes were observed for the studies where measurements were taken at least two years after the intervention. Short term negative outcomes that were found in terms of culture [34] and performance [31, 35], are not surprising: research has shown that organisations can take up to three years to recover performance back to a baseline level following the type of large structural changes required to implement hospital-wide interventions [48].

While the review design and studies assessed helped us to judge *whether* the interventions resulted in improved patient outcomes, and identified broad organisational and cultural determinants affecting the success of system-wide interventions, it was not possible to definitively explain *how* those outcomes were achieved. The quality improvement (QI) literature points to the importance of contextual factors in implementation of interventions [49, 50], and suggests that contextual factors at the microsystem level (rather than organisational factors) may have a more direct influence on QI success [51]. However, as in our study, the lack of clear and uniform definitions of organisational and other contextual factors across individual studies has hampered the ability to synthesise data [49].

### Limitations

The main limitations of this review are that only six studies were identified that met inclusion criteria, and these studies did not use common standards or measures for variables. Therefore, a meta-analysis could not be completed, and findings in relation to the impact of cultural and organisational factors on the implementation of interventions with patient outcomes could not be made.

### Implications for clinical practice and future research

Although there is a need for caution in the interpretation of our review findings, given the limited number of studies, this review highlights the need to have internationally standardised definitions of concepts such as organisational culture and climate. Standardised, validated instruments that can be applied across studies to ensure that clinical practice can be assisted by rigorous evidence-based, scientifically sound intervention studies are also required. Establishment of baseline measures for future studies is likely to be problematic, as most hospitals have been already exposed to multiple forms of patient safety or quality improvement intervention, thereby confounding any ability to attribute performance changes to specific components of an intervention.

A new way to look at the effects of previously introduced hospital-wide improvement efforts could be through reverse engineering: given the desired patient outcomes, determine how the measures of implementation would be adjusted to maintain these desired outcomes. This of course demands a thorough understanding of the underlying elements of the success and sustainability of an intervention in an acute care setting.

## Conclusion

The findings from a limited range of studies included in this review show that when implementing hospital- and system-wide interventions, there is potential for patient outcomes to be improved through changing or focusing on organisational or cultural determinants. In particular, the need for effective leadership, adequate financial and educational resources, and dedicated promotional activities appear to be common threads in the success of an intervention. The lack of standardisation of patient outcome measures – in particular, reliance on process measures and the limitations of using risk adjusted mortality as outcome measures – limit the strength of the findings. No intervention appears in more than one study or setting, therefore consistency of findings is not confirmed. Organisational interventions have a higher chance of improving patient outcomes if a wide range of staff are involved in the design, implementation and monitoring of large-scale interventions. However, changing the culture of healthcare takes time, clinical areas will adopt changes at varying paces and educational programs will have diverse effects on different groups and services.

## Electronic supplementary material

Additional file 1:**Web-accessible files include: Online appendix – table A (Search methods for identification of studies); Online appendix – table B (Study characteristics and results); and Online appendix – table C (Quality assessment of selected studies).**(DOCX 102 KB)

## References

[1] Berwick DM, Calkins DR, McCannon CJ, Hackbarth AD (2006). The 100,000 lives campaign: setting a goal and a deadline for improving health care quality. J. Am. Med. Assoc.

[2] Braithwaite J, Coiera E (2010). Beyond patient safety flatland. J R Soc Med.

[3] Donaldson L (2000). An organisation with a memory: report of an expert group on learning from adverse events in the NHS.

[4] Garling P (2008). Final report of the Special Commission of Inquiry: Acute Care Services in NSW Public Hospitals.

[5] Kohn LT, Corrigan JM, Donaldson MS (2000). To err is human: building a safer health system. A report of the Committee on Quality of Health Care in America, Institute of Medicine.

[6] Braithwaite J, Clay-Williams R, Nugus P, Plumb J: **Health care as a complex adaptive system.** In *Resilient Health Care*. Edited by: Hollnagel E, Braithwaite J, Wears R. Surrey, UK: Ashgate Publishing Limited; In press (accepted 17/10/12)

[7] Clay-Williams R: **Restructuring and the resilient organisation: implications for health care.** In *Resilient Health Care*. Edited by: Hollnagel E, Braithwaite J, Wears R. Surrey, UK: Ashgate Publishing Limited; In press (accepted 17/10/12)

[8] Braithwaite J, Westbrook M, Travaglia J (2008). Attitudes toward the large-scale implementation of an incident reporting system. Int. J. Qual. Health Care.

[9] Hillman K, Chen J, Cretikos M, Bellomo R, Brown D, Doig G, Finfer S, Flabouris A (2005). Introduction of the medical emergency team (MET) system: a cluster-randomised controlled trial. Lancet.

[10] Braithwaite J, Westbrook M, Nugus P, Greenfield D, Travaglia JF, Runciman W, Foxwell A, Boyce R, Devinney T, Westbrook J (2013). Continuing differences between health professions' attitudes: the saga of accomplishing systems-wide interprofessionalism. Int. J. Qual. Health Care.

[11] Braithwaite J, Westbrook M, Nugus P, Greenfield D, Travaglia JF, Runciman W, Foxwell A, Boyce R, Devinney T, Westbrook J (2012). A four-year, systems-wide intervention promoting interprofessional collaboration. BMC Health Serv. Res.

[12] Benn J, Burnett S, Parand A, Pinto A, Iskander S, Vincent C (2006). Studying large-scale programmes to improve patient safety in whole care systems: challenges for research. Soc. Sci. Med.

[13] Hillman K, Parr M, Flabouris A, Bishop G, Stewart A (2001). Redefining in-hospital resuscitation: the concept of the medical emergency team. Resuscitation.

[14] Sakai T, DeVita MA (2009). Rapid response system. J. Anesth.

[15] Schouten LM, Hulscher ME, Everdingen JJ, Huijsman R, Grol RP (2008). Evidence for the impact of quality improvement collaboratives: systematic review. Br. Med. J.

[16] Grimshaw JM, Eccles MP, Lavis JN, Hill SJ, Squires JE (2012). Knowledge translation of research findings. Implementation Sci.

[17] Braithwaite J, Greenfield D, Westbrook J, Pawsey M, Westbrook M, Gibberd R, Naylor J, Nathan S, Robinson M, Runciman B, Jackson M, Travaglia J, Johnston B, Yen D, McDonald H, Low L, Redman S, Johnson B, Corbett A, Hennessy D, Clark J, Lancaster J (2010). Health service accreditation as a predictor of clinical and organisational performance: a blinded, random, stratified study. Qual Saf Health Care.

[18] Landrigan CP, Parry GJ, Bones CB, Hackbarth AD, Goldmann DA, Sharek PJ (2010). Temporal trends in rates of patient harm resulting from medical care. N. Engl. J. Med.

[19] Braithwaite J (2011). A lasting legacy from Tony Blair? NHS culture change. J. R. Soc. Med.

[20] Braithwaite J, Runciman W, Merry A (2009). Towards safer, better healthcare: harnessing the natural properties of complex sociotechnical systems. Qual Saf Health Care.

[21] Braithwaite J, Westbrook M, Iedema R, Mallock N, Forsyth R, Zhang K (2005). A tale of two hospitals: assessing cultural landscapes and compositions. Soc. Sci. Med.

[22] Mannion R, Davies H, Harrison S, Frederick K, Jacobs R, Walshe K, Braithwaite J, Hyde P, Pope C (2009). Changing management cultures in the English National Heath Service. Culture and Climate in Health Care Organisations.

[23] Mannion R, Davies H, Harrison S, Konteh F, Greener I, McDonald R (2010). Quantitative explorations of culture and performance relationship: changing organisational cultures and hospital performance in the NHS.

[24] Cameron K, Freeman S (1991). Culture, congruence, strength and type: relationship to effectiveness research in organizational change and development. Res Organizational Change Dev.

[25] Nosrati H, Clay-Williams R, Cunningham F, Hillman K, Braithwaite J (2013). The role of organisational and cultural factors in the implementation of system-wide interventions in acute hospitals to improve patient outcomes: protocol for a systematic literature review. BMJ Open.

[26] Morrison A, Polisena J, Husereau D, Moulton K, Clark M, Fiander M, Mierzwinski-Urban M, Clifford T, Hutton B, Rabb D (2012). The effect of English-language restriction on systematic review-based meta-analyses: a systematic review of empirical studies. Int. J. Technol. Assess. Health Care.

[27] Webb D (2011). Safer Patients Initiative phase one. In.

[28] Webb D (2011). Safer Patients Initiative phase two. In.

[29] Grayson L, Russo P, Ryan K, Bellis K, Havers S, Heard K, Simpson P (2010). Hand Hygiene Australia Manual. In.

[30] *National Quality Forum safe practices* US: Agency for Healthcare Research and Quality; 2005.http://www.ahrq.gov/qual/30safe.htm

[31] Benning A, Dixon-Woods M, Nwulu U, Ghaleb M, Dawson J, Barber N, Franklin BD, Girling A, Hemming K, Carmalt M, Rudge G, Naicker T, Kotecha A, Derrington MC, Lilford R (2011). Multiple component patient safety intervention in English hospitals: controlled evaluation of second phase. Br. Med. J.

[32] Benning A, Ghaleb M, Suokas A, Dixon-Woods M, Dawson J, Barber N, Franklin BD, Girling A, Hemming K, Carmalt M, Rudge G, Naicker T, Nwulu U, Choudhury S, Lilford R (2011). Large scale organisational intervention to improve patient safety in four UK hospitals: mixed method evaluation. Br. Med. J.

[33] Grayson ML, Russo PL, Crulckshank M, Bear JL, Gee CA, Hughes CF, Johnson PDR, McCann R, McMillan AJ, Mitchell BG, Selvey CE, Smith RE, Wilkinson I (2011). Outcomes from the first 2 years of the Australian National Hand Hygiene Initiative. Med. J. Aust.

[34] Muething SE, Goudie A, Schoettker PJ, Donnelly LF, Goodfriend MA, Bracke TM, Brady PW, Wheeler DS, Anderson JM, Kotagal UR (2012). Quality improvement initiative to reduce serious safety events and improve patient safety culture. Pediatrics.

[35] Nowinski CJ, Becker SM, Reynolds KS, Beaumont JL, Caprini CA, Hahn EA, Peres A, Arnold BJ (2007). The impact of converting to an electronic health record on organizational culture and quality improvement. Int. J. Med. Inform.

[36] Higgins JPT, Green S, Collaboration C (2008). Cochrane handbook for systematic reviews of interventions.

[37] Larson EL, Early E, Cloonan P, Sugrue S, Parides M (2000). An organizational climate intervention associated with increased handwashing and decreased nosocomial infections. Behav. Med.

[38] Mishi S, West M (2004). Managing people and performance: an evidence-based framework applied to health service organisations. Int J Manage Rev.

[39] Zammuto RF, Krakower JK (1991). Quantitative and qualitative studies of organizational culture. Res Organizational Change Dev.

[40] Cameron KS, Quinn RE (2011). Diagnosing and changing organizational culture: based on the competing values framework.

[41] **Hospital survey on patient safety culture: items and dimensions** 2012., **2012:** http://www.ahrq.gov/qual/patientsafetyculture/hospdim.pdf

[42] Pittet D, Hugonnet S, Harbarth S, Mourouga P, Sauvan V, Touveneau S, Perneger TV (2000). Effectiveness of a hospital-wide programme to improve compliance with hand hygiene. Infection Control Programme. Lancet.

[43] Lilford R, Pronovost P (2010). Using hospital mortality rates to judge hospital performance: a bad idea that just won’t go away. Br Med J.

[44] Pitches DW, Mohammed MA, Lilford RJ (2007). What is the empirical evidence that hospitals with higher-risk adjusted mortality rates provide poorer quality care? A systematic review of the literature. BMC Health Serv. Res.

[45] Wolosin R (1997). Press Ganey Satisfaction Measurement, Client Reference Manua.

[46] Dixon-Woods M, Bosk CL, Aveling EL, Goeschel CA, Pronovost PJ (2011). Explaining Michigan: developing an ex post theory of a quality improvement program. Milbank Q.

[47] Santamaria J, Tobin A, Holmes J (2010). Changing cardiac arrest and hospital mortality rates through a medical emergency team takes time and constant review. Crit. Care Med.

[48] Braithwaite J, Westbrook J, Iedema R (2005). Restructuring as gratification. J. R. Soc. Med.

[49] Kaplan HC, Brady PW, Dritz MC, Hooper DK, Linam W, Froehle CM, Margolis P (2010). The influence of context on quality improvement success in health care: a systematic review of the literature. Milbank Q.

[50] Van Herck P, De Smedt D, Annemans L, Remmen R, Rosenthal MB, Sermeus W (2010). Systematic review: effects, design choices, and context of pay-for-performance in health care. BMC Health Serv Res.

[51] Kaplan HC, Provost LP, Froehle CM, Margolis PA (2012). The Model for Understanding Success in Quality (MUSIQ): building a theory of context in healthcare quality improvement. BMJ Qual Saf.

[CR52] The pre-publication history for this paper can be accessed here:http://www.biomedcentral.com/1472-6963/14/369/prepub

