# Supplementary material for: Do large-scale hospital- and system-wide interventions improve patient outcomes: a systematic review
Source: BMC Health Serv Res. 2014 Sep 3;14:369. doi: 10.1186/1472-6963-14-369 (PMC4282191; doi:10.1186/1472-6963-14-369)
Supplement: Supplementary file 1 — Additional file 1:Web-accessible files include: Online appendix – table A (Search methods for identification of studies); Online appendix – table B (Study characteristics and results); and Online appendix – table C (Quality assessment of selected studies).(DOCX 102 KB) [file 12913_2013_3695_MOESM1_ESM.docx]

**Appendices and supplementary material**

Web-accessible files include: Online appendix – table A (Search methods for identification of studies); Online appendix – table B (Study characteristics and results); and Online appendix – table C (Quality assessment of selected studies).

Online Table A: Search methods for identification of studies

| Database | Search Terms |
| --- | --- |
| Medline,Pre-Medline | organi?ational culture.sh. OR organi?tion$ culture$*.tw. OR organi?ation$ climate*.tw. OR organi?ation$ context*.tw. OR organi?ation$ characteristic*.tw. OR work$ culture*.tw. OR corporate$ culture*.tw. OR organi?ation$ goal*.tw. OR organi?ation$ value*.tw.  AND  adopt$ adj2 organi?ation*).tw OR (adherence adj2 protocol*).tw OR (organizational innovation/) OR (“diffusion of innovation”/) OR ($innovation) .tw OR ($intervention.tw) OR ($diffusion.tw) OR (organi?ation$ change*) OR (protocol$ change*) OR(practice$ change*) OR (structure$ change*) OR ($adoption.tw) OR ($leader$).tw OR (intervention.sh) OR (adoption/eh,px,st[ethnology,Psycology,Standards])  AND  patient$ adj2 outcome*  AND  Health$ organi?ation* OR hospital* OR health$ facilit$ |
| CINAHL | MH Organizational Culture OR TX Organi?ation* N5 Cultur* OR Organi?ation* characteristic* OR TX organi?ation* climate* OR TX organi?ation* context* OR TX work* culture* OR TX corporate* culture* OR TX organi? ation* value* OR TX organi?ation* goal*  AND  MH diffusion of innovation OR TX adopt* N2 organi?ation* OR TX adherence N2 protocol* OR TX innovation OR TX diffusion OR TX organi?ation* N2 change* OR TX protocol* N2 change* OR TX practice* N2 change* OR TX structure* N2 change* OR TX adoption OR TX leader* OR TX intervention  AND  TX patient* N2 outcome*  AND  TX health$ organi?ation* OR TX hospital# OR TX health* facilit* |
| EMBASE | ((organi?ation$ adj5 cultur$) OR organi?ation$ characteristic$ OR organi?ation$ climate$ OR organi?ation$ context$ OR work$ culture$ OR corporate culture$ OR organi?ation$ value$ OR organi?ation$ goal$).tw.  AND  ($innovation OR $diffusion OR $intervention OR organi?ation$ change$ OR protocol$ change$ OR practice$ change$).tw. OR (adopt$ adj2 organi?ation$).tw. OR adherence adj2 protocol$).tw. OR (structure$ change$ OR $adoption tw OR $leader$ tw).tw. OR $adoption.sh.  AND  patient$ adj2 outcome$  AND  (health$ organi?ation* OR hospital* OR health$ facilit$).tw. |
| WEB OF SCIENCE | TS = (corporate culture* OR work* culture* OR organi?ation* climate* OR organi?ation* value* OR organi?ation* goal* OR organi?ation* characteristic* OR organi?ation* structure*) OR TS =(organi?ation* SAME culture*)  AND  TS =( (hospital*) OR (health care organi?ation) OR (health facilit*) OR (health care facilit*))  AND  TS = (patient* SAME outcome*)  AND  TS = (diffusion of innovation OR adopt* organi?ation* OR innovation* OR intervention* OR diffusion OR organi?ation* change* OR protocol change* OR practice* change* OR structure change* OR adoption OR leader* OR intervention) |
| PsycINFO | (exp Organizational Climate/ ) OR (organi?ation$ culture* OR organi?ation$ characteristic* OR organi?ation$ structure* OR corporate culture* OR work$ culture* OR organi?ation$ context* OR organi?ation$ value* OR organi?ation$ goal*).ab. OR (organi?ation$ culture* OR organi?ation$ characteristic* OR organi?ation$ structure* OR corporate culture* OR work$ culture* OR organi?ation$ context* OR organi?ation$ value* OR organi?ation$ goal*).ti.  AND  exp Intervention/ OR exp Innovation/ OR (adopt$ adj2 organi?ation*).ab. OR (adherence adj2 protocol*).ab. OR (adopt$ adj2 organi?ation*).ti. OR (adherence adj2 protocol*).ti.OR (intervention OR diffusion OR organi?ation$ change* OR protocol$ change* OR practice$ change* OR structure$ change* OR adoption OR $leader$).ab. OR (intervention OR diffusion OR organi?ation$ change* OR protocol$ change* OR practice$ change* OR structure$ change* OR adoption OR $leader$).ti.  AND  (Health$ organi?ation* OR hospital* OR health$ facilit$).ti.OR (Health$ organi?ation* OR hospital* OR health$ facilit$).ab.  AND  (Patient$ adj2 outcome*).ab. OR (patient$ adj2 outcome*).ti. |
| Scopus | TITLE-ABS-KEY (organi?ational culture* OR organi?ation$ characteristic* OR organi?ation$ climate* OR organi?ation$ context* OR work$ culture* OR corporate$ culture* OR organi?ation$ value* OR organi?ation$ goal*)  AND  (intervention OR innovation OR diffusion OR protocols)  AND  ("Health$ organi?ation*" OR "hospital*" OR "health$ facilit$")  AND  (patient* outcome*)) |
| Global Health | (organi?ational culture* OR organi?ation$ characteristic* OR organi?ation$ climate* OR organi?ation$ context* OR work$ culture* OR corporate$ culture* OR organi?ation$ value* OR organi?ation$ goal*).ab. OR (organi?ational culture* OR organi?ation$ characteristic* OR organi?ation$ climate* OR organi?ation$ context* OR work$ culture* OR corporate$ culture* OR organi?ation$ value* OR organi?ation$ goal*).ti.  AND  innovations/ OR innovation adoption/ OR adoption/ OR (adopt$ adj2 organi?ation*).ab. OR (adopt$ adj2 organi?ation*).ti. OR (adherence adj2 protocol*).ab. OR ($innovation OR $intervention OR $diffusion OR organi?ation$ change* OR protocol$ change* OR practice$ change* OR structure$ change* OR $adoption OR $leader$).ab. OR ($innovation OR $intervention OR $diffusion OR organi?ation$ change* OR protocol$ change* OR practice$ change* OR structure$ change* OR $adoption OR $leader$).ti.  AND  (patient$ adj2 outcome*).ab. OR (patient$ adj2 outcome*).ti.(Health$ organi?ation* OR hospital* OR health$ facilit$).ti. OR (Health$ organi?ation* OR hospital* OR health$ facilit$).ab. |

Online Table B: Study characteristics and results

**Larson et.al. 2000**

| Duration and follow-up | Intervention three months, follow-up six months later. |
| --- | --- |
| Setting and study design | Two hospitals in mid-Atlantic region, one as intervention site and the other as comparison; 250 beds each.  Non-randomised controlled trial. |
| Aim | To assess the impact of an intervention to change organisational culture on the frequency of staff handwashing and on the incidence of nosocomial infections associated with Methicillin-Resistant Staphylococcus Aureus (MRSA) and Vancomycin-Resistant Enterococci (VRE). |
| Method | Three phase intervention, consisting of baseline, implementation and follow-up. Frequency of staff handwashing determined by counting devices inserted into soap dispensers in four critical units Infection data collected by infection control staff in each hospital as part of their usual routine. Surveillance methods were the same in both hospitals. Laboratory-based surveillance procedures and definitions developed by the Centers for Disease Control and Prevention (CDC) used by hospitals participating in the National Nosocomial Infections Surveillance System were applied to determine whether an infection was present and was community-acquired or nosocomial. No routine screening cultures were obtained in either hospital. |
| Hospital-wide intervention | Administrative intervention handwashing. |
| Organisational factors | Commitment and support from top management, medical and nursing leaders; Involving all-levels in design, implementation and monitoring the procedure. |
| Process outcomes | The number of activations of soap dispensers in two study units in each hospital per 1000 patient-care days. |
| Patient outcomes | Rates of nosocomial infections associated with MRSA and VRE, reported as number of infections per 1000 patient-care days. |
| Findings | In the study hospital, the mean handwashing frequency per patient-care day at six month follow up was double that of the comparison hospital.  In the baseline comparison there were no significant difference between rates of VRE and MRSA in study and control hospitals.  The study hospital showed significant lower rates in terms of VRE both in implementation and follow-up phases, but no significant differences in MRSA in those phases.  The ratio of change (i.e. the reduction in infection rates) in the intervention hospital between baseline and follow up phases for MRSA and VRE were both significantly greater than the ratios of change in comparison hospital (p<0.0001). |

**Nowinski et.al. 2007**

| Duration and follow-up | Intervention one year and four months, follow-up one year later. |
| --- | --- |
| Setting and study design | Non-profit, integrated healthcare delivery system comprised of three hospitals (with a total bed count of approximately 850 beds).  Observational study. |
| Aim | Evaluating changes in organisational culture and quality during and subsequent to conversion to an electronic health record (EHR). |
| Method | The study made use of both survey data collected specifically for the purposes of the project (primary data) and ongoing corporate data collection activities (secondary data). The Culture and Quality Questionnaire (CQQ), the primary data tool, was sent via interoffice mail to all employees holding a managerial position or higher (including employed physicians) in December 2002, prior to conversion to the EHR in order to obtain baseline data. 621 employees completed the baseline survey (54% response rate) and 471 completed the follow-up survey (38% response rate). |
| Hospital-wide intervention | System-wide single-vendor electronic health record. |
| Organisational factors | Organisational culture: Group, Developmental, Hierarchical, and Rational. |
| Process outcomes | Continuous Quality Improvement maturity (CQI) scores in terms of leadership, customer satisfaction, quality management, information and analysis, quality results, human resource utilisation, and strategic quality planning. |
| Patient outcomes | Quality Improvement (QI) indicators:   1. Initial antibiotic dose within 4h of hospital arrival for pneumonia patients 2. Fall rate per 1000 patient-days 3. Chest pain pathway-discharged within 23h of admission 4. Annual HgA1c measurement in diabetic patients 5. Left ventricular function evaluation on a yearly basis 6. Appropriate use/non-use of ACE inhibitors at discharge for patients with CHF 7. Patient Satisfaction (measured through Press Ganey survey) |
| Findings | Least-squares adjusted means for group culture decreased from 21.8 to 20.0; the least-squares adjusted means for hierarchical culture increased from 30.0 to 31.9 after 12 months (change only significant in one of five hospitals for group culture and two of five hospitals for hierarchical culture). Least-squares adjusted means for leadership showed decrease in the leadership scale after 12 months of electronic health record implementation from 3.63 to 3.54, but this was only significant (p<0.05) in one of five hospitals.  There were several strong (>0.94) correlations between changes in culture scores and changes in quality indicators at the three acute care facilities. Appropriate discharge of patients with chest pain was negatively correlated with developmental culture; use of antibiotics within 4h of admission was positively associated with rational culture and quality management and negatively related to group culture and human resource utilisation; and patient satisfaction was positively correlated with group culture and negatively correlated with rational culture.  Decrease of 16% in CQI for initial antibiotic dose within 4h of hospital arrival for pneumonia patients (p<0.001) between intervention and follow-up. Decrease of 3% in CQI for chest pain pathway-discharged within 23h of admission (p<0.023) for one of three hospitals between intervention and follow-up. Decreased patient satisfaction for two of three hospitals between intervention and follow-up (1%, p<0.003 and 2%, p<0.019). |

**Grayson et.al. 2011**

| Duration and follow-up | Intervention one year, follow-up two years later. |
| --- | --- |
| Setting and study design | 512 Australian hospitals.  Observational study. |
| Aim | To report outcomes from the first 2 years of the National Hand Hygiene Initiative (NHHI), a hand hygiene (HH) culture-change program implemented in all Australian hospitals to improve health care workers’ HH compliance, increase use of alcohol-based hand rub and reduce the risk of health care-associated infections. |
| Method | Staphylococcus aureus bacteraemia (SAB) infections from study hospitals in all states and territories were collated two years before (2007-2008) and two years after (2009-2010) implementation of the NHHI. |
| Hospital-wide intervention | The Australian National Hand Hygiene Initiative (NHHI). |
| Organisational factors | Leadership, education and training, promotion and awareness of the intervention, engaging senior executives and clinicians. Introduction of a standardised auditing tool, the ‘5 Moments’ of Hand Hygiene. |
| Process outcomes | Hand-hygiene compliance. |
| Patient outcomes | SAB incidence rates. The national rate per month was used (patient with SAB as numerator and either patient days or occupied bed days as denominator). |
| Findings | In late 2010, overall national HH compliance rate in 521 hospitals was 68.3% (168 641/246 931 moments), but HH compliance before patient contact was 10%–15% lower than after patient contact. Among sites new to the ‘5 Moments’ audit tool, HH compliance improved from 43.6% (6431/14740) at baseline to 67.8% (106 851/157 708) (p<0.001).  Educational program had different effects on various groups of healthcare workers. HH compliance was highest among nursing staff (73.6%; 116 851/158 732) and worst among medical staff (52.3%; 17 897/34 224) after 2 years.   1. National incidence rates of methicillin resistant SAB were stable for the 18 months prior to NHHI (Jul 2007-2008; p=0.366) but declined after implementation (2009-2010; p=0.008). |

**Benning et.al. 2011**

| Duration and follow-up | Intervention 18 months, follow-up of sub-study3 after 20 months, follow-up of sub-study4 after 6 months. |
| --- | --- |
| Setting and study design | Four hospitals (one in each country in the UK) participating in the first phase of the SPI (SPI1); 18 control hospitals.  Controlled mixed method evaluation involving five sub-studies, before and after design. |
| Aim | Independent evaluation of the first phase of the Health Foundation's Safer Patients Initiative (SPI) to determine generic aspects of SPI and to identify the net additional effect of SPI and any differences in changes in participating and non-participating NHS hospitals. SPI1 sought to reduce adverse events by 50%. |
| Method | Sub-study1. interviews with strategic/senior staff consisting of semi-structured telephone interviews with 60 hospital staff members in strategic/senior positions across the four hospitals, seeking information on how far participants understood and expressed enthusiasm for the SPI1.  Sub-study2. The National NHS Staff Survey questionnaire was used at two time points to measure variables such as staff morale, attitudes, and aspects of “culture” that might be affected by the generic strengthening of organisational systems that SPI1 intended to achieve. Eleven of the 28 survey questions were identified as likely to be of relevance. Questionnaires were sent to all staff members in the four SPI1 hospitals, including the three hospitals outside England.  Sub-study3. Qualitative study consisting of three rounds of data collection. First round, 150 hours of ethnographic observations and 47 interviews with different types of ward staff, the second round, 150 hours ethnographic observations and 41 interviews, the third round three focus groups at each site (one study at ward level, one involving people with responsibilities for patient safety/SPI1, and one at strategic level) was used to feed back preliminary findings and to ask staff for their reflections on SPI1.  Sub-study4. Quality of care of patients aged>65 with acute respiratory disease, case note reviews (both explicit and holistic).  Sub-study5. Outcomes:   1. Adverse events in patients aged >65 with acute respiratory disease through holistic case note review. 2. Hospital mortality in patients aged >65 with acute respiratory disease through case note review. 3. Patient satisfaction through questionnaire as used in NHS patient surveys. |
| Hospital-wide intervention | First phase of the Health Foundation’s Safer Patients  Initiative (SPI): The SPI1 was a multi-faceted organisational intervention. |
| Organisational factors | Staff morale and opinion, organisational climate. |
| Process outcomes | Senior staff interviews (sub-study 1), General staff questionnaires (sub-study 2), Ethnographic observations on wards (sub-study 3), quantification of error rates (sub-study 4). |
| Patient outcomes | 1. Adverse events. 2. Mortality rate. 3. Patient satisfaction. |
| Findings | Introduction of SPI1 associated with improvements in one of the types of clinical process studied (monitoring of vital signs) and one measure of staff perceptions of organisational climate (p<0.01). One of the patient satisfaction scores (cleanliness of the bathrooms) improved in the intervention hospitals. There was no additional effect of SPI1 on other targeted issues nor on other measures of generic organisational strengthening. |

**Benning et.al. 2011**

| Duration and follow-up | Intervention 20 months, follow up of sub-study1 after three months; follow up of sub-studies2 and 3 after six months; follow up of sub-study4 concurrent with intervention; follow up of sub-study5: mortality rate in ICU: on a monthly basis up to six months after the intervention; the infection rate outcome: Data on C difficile were available on a three monthly basis up to nine months after the intervention, MRSA data were available every three months up to a year after the intervention, and patient outcome: three months after the intervention |
| --- | --- |
| Setting and study design | 18 UK hospitals, 9 hospitals for intervention and 9 matched control sites with similar number of beds, same area (rural or urban).  Controlled mixed method evaluation involving five sub-studies, before and after design.. |
| Aim | Independent evaluation of the second phase of the Health Foundation's Safer Patients Initiative (SPI) with aim to reduce adverse events by 30% and hospital mortality by 15%. |
| Method | Sub-study1. Staff survey of staff morale, culture, and opinion using NHS national staff survey, 850 staff members per site.  Sub-study2. Quality of care study assessing acute medical care in patients aged >65 with acute respiratory disease, using15 sets of case note reviews (from each control and SPI2 hospital).  Sub-study3. Quality of preoperative care study assessing perioperative care in patients with total hip replacement and open colectomy, using explicit case note review.  Sub-study 4. Clinical process measures including usage of consumables for hand hygiene, data gathered using national observation study of effectiveness of national “cleanyourhands” campaign, monthly data were collected from NHS Logistics on consumption of soap and alcohol hand rub (as an indirect measure of compliance with hand hygiene).  Sub-study 5. Outcomes:   1. Adverse events in patients aged >65 with acute respiratory disease, using holistic case note review. as per the evaluation of SPI1. 2. Hospital mortality in patients aged >65 with acute respiratory disease, using case note review. 3. Intensive care unit mortality using routine data from intensive care national audit and research centre. 4. Infection rates associated with healthcare using routine data from Health Protection Agency. 5. Patient satisfaction using NHS patient surveys (as per SPI1). |
| Hospital-wide intervention | Second phase of the Health Foundation’s Safer Patients Initiative (SPI) the SPI2 was a multi-faceted organisational intervention delivered in 18 UK hospitals. |
| Organisational factors | 1. Working in well-structured teams. 2. Work related stress in previous 12 months. 3. Staff job satisfaction. 4. Quality of work-life balance. 5. Support from supervisors. 6. Organisational climate. |
| Process outcomes | 1. Monitoring of vital signs. 2. Adherence rates for four perioperative standards. 3. Prescribing errors. 4. Medical history (exercise tolerance and occupation). 5. Consumption of soap and alcohol for handwashing. |
| Patient outcomes | 1. Adverse events. 2. Mortality among acute respiratory patients. 3. Mortality among patients in intensive care units. 4. Rates of infection with C difficile per 1000 and MRSA per 100 000 bed occupancy days. 5. Patient satisfaction. |
| Findings | Significant decrease in one measure of staff perception of organisational climate (p<0.01). Many aspects of care were already good or improving across the NHS in England, suggesting considerable improvements in quality across the board. Improvements probably due to contemporaneous policy activities relating to patient safety, including those with features similar to the SPI, and the emergence of professional consensus on some clinical processes. This phenomenon may have attenuated the incremental effect of the SPI, making it difficult to detect. Alternatively, the full impact of the SPI might be observable only in the longer term. The conclusion of this study could have been different if concurrent controls had not been used. |

**Muething et.al. 2012**

| Duration and follow-up | Intervention commenced 2006, intervention and data collection ongoing. |
| --- | --- |
| Setting and study design | Cincinnati Children's Hospital Medical Centre (CCHMC), a large urban paediatric academic medical centre, 32000 in-patient admissions.  Observational study. |
| Aim | Reducing Serious Safety Events (SSEs) to 0.2 per 10 000 adjusted patient-days by June 30, 2010. |
| Method | A multidisciplinary SSE reduction team reviewed the safety literature, examined recent SSEs, reviewed results of HSPSC survey, interviewed internal leaders, and visited other leading organisations. Senior hospital leaders provided oversight, monitored progress, and helped to overcome barriers. Interventions focused on: (1) error prevention; (2) restructuring patient safety governance; (3) a new root cause analysis process and a common cause database; (4) a highly visible lessons learned program; and (5) specific tactical interventions for high-risk areas. Primary outcome measure was the rate of SSEs and secondary outcome measure was the change in patient safety culture. |
| Hospital-wide intervention | Cultural and system changes to improve patient safety:  1. Error prevention.  2. Restructuring patient safety governance.  3. Root cause analysis.  4. Lessons learned program specific tactical interventions for high-risk areas. |
| Organisational factors | Safety culture dimension (unit level):  1. Supervisor and /managers expectations and actions promoting patient safety.  2. Organisational learning, continuous improvement.  3. Teamwork within hospital units.  4. Communication openness.  5. Feedback and communication about error.  6. Non-punitive response to error.  7. Staffing. Safety culture dimension (hospital level):  1. Hospital management support for patient safety.  2. Teamwork across hospital units.  3. Hospital handoffs and transitions. |
| Process outcomes | Patient safety culture. |
| Patient outcomes | 1. Rate of serious safety events (SSEs).  2. Days between SSEs. |
| Findings | Following the intervention, SSEs per 10,000 adjusted patient days significantly decreased from a mean of 0.9 to 0.3 (p<0.0001). Days between SSEs increased from a mean of 19.4 to 55.2 (p<0.0001). During initial phase of the intervention, results from the safety culture survey worsened. However, as the initiative progressed, there was improvement. Individual clinical areas adopted changes at varying paces, so persistence over time was vital to achieving the overall organisational goal. Survey response rates increased over time from 17.8% in 2005 to 31.3% in 2009.. |

Online Table C: Quality assessment of selected studies

| **Study** | **Selection bias** | **Blinding to assessor** | **Protection against contamination** | **Reliable outcome measures** | **Was a control group used?** | **Was risk adjustment or a comparison of baseline characteristic reported** | **Statistical analysis undertaken** |
| --- | --- | --- | --- | --- | --- | --- | --- |
| Larson 1998[^1^](#_ENREF_1) | Unclear (method for selection of study hospital not indicated) | Partial (qual data collection not blinded) | Low risk of contamination (separate study and control hospitals) | Yes | Yes | Yes | Yes |
| Grayson 2011[^2^](#_ENREF_2) | Unclear (no control, attrition not stated) | Not applicable | Not addressed | Yes | No | Yes | Yes |
| Nowinski 2007[^3^](#_ENREF_3) | Unclear (no control, attrition not stated), | Partial (qual data collection not blinded) | Not addressed | Mostly (survey measures self assessed) | No | Yes | Yes |
| Benning 2011[^4^](#_ENREF_4) | Moderate (study hospitals positively selected based on factors likely to lead to program success, although adjustments made for baseline differences) | Partial (observers were independent, but observers, researchers and study respondents not blinded, although most results null) | High risk of contamination overall with other improvements occurring across health services | Yes | Yes | Yes | Yes |
| Benning  2011[^5^](#_ENREF_5) | Moderate (study hospitals positively selected based on factors likely to lead to program success, although adjustments made for baseline differences) | Partial (researchers were independent, but researchers and study respondents not blinded, although most results null) | High risk of contamination overall with other improvements occurring across health services | Yes | Yes | Yes | Yes |
| Muething 2012[^6^](#_ENREF_6) | Unclear (no control, attrition not stated) | Partial (qual data collection not blinded) | Moderate risk of contamination with other ongoing improvement processes | Yes | No | Yes | Yes |

**References**

1. Larson EL, Early E, Cloonan P, Sugrue S, Parides M. An organizational climate intervention associated with increased handwashing and decreased nosocomial infections. *Behavioral Medicine* 2000;26(1):14-22.

2. Grayson ML, Russo PL, Crulckshank M, Bear JL, Gee CA, Hughes CF, et al. Outcomes from the first 2 years of the Australian National Hand Hygiene Initiative. *Medical Journal of Australia* 2011;195(10):615-19.

3. Nowinski CJ, Becker SM, Reynolds KS, Beaumont JL, Caprini CA, Hahn EA, et al. The impact of converting to an electronic health record on organizational culture and quality improvement. *International Journal of Medical Informatics* 2007;76 Suppl 1:S174-83.

4. Benning A, Ghaleb M, Suokas A, Dixon-Woods M, Dawson J, Barber N, et al. Large scale organisational intervention to improve patient safety in four UK hospitals: mixed method evaluation. *Brtish Medical Journal* 2011;342:d195.

5. Benning A, Dixon-Woods M, Nwulu U, Ghaleb M, Dawson J, Barber N, et al. Multiple component patient safety intervention in English hospitals: controlled evaluation of second phase. *British Medical Journal* 2011;342:d199.

6. Muething SE, Goudie A, Schoettker PJ, Donnelly LF, Goodfriend MA, Bracke TM, et al. Quality improvement initiative to reduce serious safety events and improve patient safety culture. *Pediatrics* 2012;130(2):e423-31.
